# Detecting inbreeding depression for reproductive traits in Iberian pigs using genome-wide data

**DOI:** 10.1186/s12711-014-0081-5

**Published:** 2015-01-17

**Authors:** María Saura, Almudena Fernández, Luis Varona, Ana I Fernández, Maria Ángeles R de Cara, Carmen Barragán, Beatriz Villanueva

**Affiliations:** Departamento de Mejora Genética Animal, INIA, Carretera de la Coruña km 7.5, 28040 Madrid, Spain; Unidad de Genética Cuantitativa y Mejora Animal, Facultad de Veterinaria, Universidad de Zaragoza, Miguel Servet 177, 50013 Zaragoza, Spain; Laboratoire d’Eco-anthropologie et Ethnobiologie, UMR 7206 CNRS/MNHN/Université Paris 7, Muséum National d’Histoire Naturelle, CP 139, 57 rue Cuvier, F-75231 Paris, France

## Abstract

**Background:**

The current availability of genotypes for very large numbers of single nucleotide polymorphisms (SNPs) is leading to more accurate estimates of inbreeding coefficients and more detailed approaches for detecting inbreeding depression. In the present study, genome-wide information was used to detect inbreeding depression for two reproductive traits (total number of piglets born and number of piglets born alive) in an ancient strain of Iberian pigs (the Guadyerbas strain) that is currently under serious danger of extinction.

**Methods:**

A total of 109 sows with phenotypic records were genotyped with the PorcineSNP60 BeadChip v1. Inbreeding depression was estimated using a bivariate animal model in which the inbreeding coefficient was included as a covariate. We used two different measures of genomic inbreeding to perform the analyses: inbreeding estimated on a SNP-by-SNP basis and inbreeding estimated from runs of homozygosity. We also performed the analyses using pedigree-based inbreeding.

**Results:**

Significant inbreeding depression was detected for both traits using all three measures of inbreeding. Genome-wide information allowed us to identify one region on chromosome 13 associated with inbreeding depression. This region spans from 27 to 54 Mb and overlaps with a previously detected quantitative trait locus and includes the *inter-alpha-trypsin inhibitor* gene cluster that is involved with embryo implantation.

**Conclusions:**

Our results highlight the value of high-density SNP genotyping for providing new insights on where genes causing inbreeding depression are located in the genome. Genomic measures of inbreeding obtained on a SNP-by-SNP basis or those based on the presence/absence of runs of homozygosity represent a suitable alternative to pedigree-based measures to detect inbreeding depression, and a useful tool for mapping studies. To our knowledge, this is the first study in domesticated animals using the SNP-by-SNP inbreeding coefficient to map specific regions within chromosomes associated with inbreeding depression.

**Electronic supplementary material:**

The online version of this article (doi:10.1186/s12711-014-0081-5) contains supplementary material, which is available to authorized users.

## Background

The reduction in performance due to inbreeding (i.e. inbreeding depression) has long been documented in plant and animal populations [1]. In general, inbreeding depression is most severe for traits that are closely related with fitness, but other traits can also be affected [2]. The standard approach for estimating inbreeding depression is to regress the phenotype of the trait of interest on the inbreeding coefficient (*F*). Typically, *F*, defined as the probability that both alleles at any locus within an individual are identical by descent (IBD), has been computed from pedigree information. However, the current availability of very large numbers of single nucleotide polymorphisms (SNPs) offers new opportunities to improve the accuracy of *F* estimates [3] and to develop more detailed approaches for detecting inbreeding depression [4-6]. Several potential advantages of using genomic *F* rather than pedigree-based *F* (*F*_*ped*_) have been highlighted [4,5]. Genomic *F* measures homozygosity directly and thus can more accurately reflect the actual percentage of the genome that is homozygous, whereas *F*_*ped*_ is only an expectation of that percentage. Another critical difference is that genomic *F* allows us to estimate inbreeding and inbreeding depression for specific genomic regions, which is not possible with *F*_*ped*_. In addition, genomic *F* can be estimated in populations where pedigree recording is difficult or impossible.

Several alternative estimates of genomic *F* based on SNP genotypes have been proposed. A simple estimate can be obtained on a SNP-by-SNP basis as the proportion of homozygous genotypes [3-6]. However, the drawback of this estimate is that it does not differentiate between IBD and identity by state (IBS). An alternative approach for quantifying individual homozygosity that better reflects IBD is based on runs of homozygosity (ROH). The idea is that autozygous genotypes are not evenly distributed throughout the genome but are distributed in runs that are inherited together [7,8]. This is explained by consanguineous matings causing inheritance of haplotypes that are IBD and result in homozygous stretches along the genome of the offspring [9]. It has been shown that these runs provide a good measure of individual genome-wide autozygosity (*F*_*roh*_) and allow us also to distinguish between recent and ancient inbreeding [10].

The aim of this study was to detect genomic regions responsible for inbreeding depression for two reproductive traits in a highly inbred strain of Iberian pigs (the Guadyerbas strain) using different measures of genomic *F*.

## Methods

### Animals, pedigree and phenotypic data

In this study, data originated from Guadyerbas pigs maintained in a small isolated herd at the CIA ‘El Dehesón del Encinar’ (Oropesa, Toledo, Spain). Four males and 20 females founded the herd that has been maintained in isolation under a genetic conservation program [11]. Complete and very accurate genealogy is available since the foundation of the herd in 1944. It comprises about 25 generations and includes 1178 animals born in the herd between 1947 and 2011. The effective population size (*N*_*e*_) estimated from the rate at which pedigree-based or SNP-based coancestry increases has been estimated to be about 10 [3].

Phenotypic data for total number of piglets born (TNB) and number of piglets born alive (NBA) in successive parities from pedigreed sows were available. Means (standard deviations) for TNB and NBA were 7.39 (2.34) and 7.06 (2.25), respectively. Farrowing facilities were improved in 2000 with a new building where piglets had *ad libitum* access to creep food from seven days of age onwards. No creep food was supplied before 2000. Thus, there were eight levels of management by combining season of farrowing (four seasons) and farrowing facilities (two). Boars from two strains of Iberian pigs (Guadyerbas and Torbiscal) were used in the matings. Offspring fathered by Torbiscal boars were never maintained in the herd.

### Genotyping data

All animals born in the herd between 1992 and 2011 (about six generations) were genotyped. They included 86 males and 141 females. Of these females, 113 had phenotypic records for litter size. Genomic DNA extracted from blood samples was hybridized with the Illumina PorcineSNP60 BeadChip v1 and images were scanned by an external service (Universidad Autónoma de Barcelona, Spain). The SNP chip comprises 62 163 probes that are distributed across 18 autosomes and the two sex chromosomes according to the latest version of the porcine gene annotation (*Sscrofa*10.2). Genotype calls were obtained with the Genotyping Module of the GenomeStudio Data Analysis software (Illumina Inc.). For the purpose of increasing the power of genotype calling (i.e. to correctly determine the genotype for each individual at each SNP), we included samples from other strains of Iberian pigs. In total, 468 genotyped samples were used in this step. These comprised the 227 Guadyerbas samples (including the 113 sows with phenotypic records) and 241 samples from other strains. The extra samples were not used in any further analysis.

Quality control procedures were applied to identify problematic SNPs and samples. First, SNPs that did not satisfy the following quality control criteria were removed: Call Frequency < 0.99, GenTrainScore < 0.70, AB R Mean < 0.35 and number of inconsistencies with the genealogy > 9 (see Saura et al. [3] for further details on the filtering criteria performed). Unmapped SNPs and SNPs mapped to sex chromosomes were also excluded. A total of 51 127 SNPs remained and were used in subsequent analyses. Note that monomorphic SNPs (i.e., those with a minor allele frequency (MAF) of 0 were not removed. After filtering SNPs, the data were reanalysed and samples with a Call Rate < 0.96 and with a large number of inconsistencies with the genealogy were removed. Four samples were excluded, so the final number of genotyped Guadyerbas females available was 109. The total number of litter records from these 109 genotyped sows was 265.

### Inbreeding coefficients

Different estimates of *F* for the sows were used for the inbreeding depression analyses:

1. Genealogical inbreeding coefficients (*F*_*ped*_) were obtained using all pedigree information that had been recorded since the foundation of the herd.
2. Genomic SNP-by-SNP inbreeding coefficients (*F*_*snp*_) were obtained based on the excess of SNP homozygosity, as in Keller et al. [4]. The inbreeding coefficient for individual *i* (*F*_*snp(i)*_) was computed as *F*_*snp*(*i*)_ = [(*OH*_*i*_ − *EH*)/(*s* − *EH*), where *s* is the number of SNPs, *OH*_*i*_ is ${\displaystyle {\sum}_{j=1}^s{X}_{ij},}$ where *X*_*ij*_ is an indicator variable taking values of 1 if individual *i* is homozygous for SNP *j*, and 0 if individual *i* is heterozygous for SNP *j*, and *EH* is the expected homozygosity in the population. The expected homozygosity was computed as ${\displaystyle {\sum}_{j=1}^s\left[1-2{p}_j\left(1-{p}_j\right)\right],}$ where *p*_*j*_ is the MAF for SNP *j*. We also computed SNP-by-SNP inbreeding coefficients as the proportion of SNPs that are homozygous for the individual (*F*_*snp_r*_). Note that *F*_*snp_r*_ ranges from 0 to 1 but *F*_*snp*_ can be negative (when *EH* is higher than *OH*_*i*_).
3. Genomic inbreeding coefficients were also estimated based on ROH (*F*_*roh*_). For a given individual *i*, *F*_*roh(i)*_ was defined as the proportion of its genome that is in ROH [12]. We used our own Fortran code to detect ROH [13] that were defined using the following criteria: (i) a maximum of two missing genotypes and one heterozygous genotype were permitted in a ROH; (ii) the minimum SNP density required to define a ROH was 1 SNP per 100 kb; (iii) the maximum distance allowed between two consecutive homozygous SNPs in a ROH was 1 Mb; and (iv) the minimum number of SNPs that constitute a ROH was 30. We also performed analyses based on short and long ROH. We defined the inbreeding coefficient based on short ROH for individual *i* (*F*_*roh_short(i)*_) as the proportion of its genome that was in ROH of lengths between 0.5 and 5 Mb and the inbreeding coefficient based on long ROH (*F*_*roh_long(i)*_) as the proportion of its genome that is in ROH of lengths > 5 Mb. These thresholds were applied to assess the relative importance of distant (*F*_*roh_short*_) *versus* recent (*F*_*roh_long*_) inbreeding [13]. Long ROH are expected to be autozygous segments that originated from recent common ancestors, while short ROH are likely to have originated from more remote common ancestors [9].

### Inbreeding depression analyses

Inbreeding depression was estimated by regressing the phenotype of the reproductive trait (TNB and NBA) on *F*. This regression was performed by including *F* as a covariate in a bivariate animal model analysis. The model equation for both traits was:

$$ \mathbf{y}=\mathbf{X}\beta +{\mathbf{Z}}_{\mathbf{1}}\mathbf{a}+{\mathbf{Z}}_{\mathbf{2}}\mathbf{p}+\mathbf{e}, $$

where **y** is the vector of observations for TNB or NBA, ***β*** is the vector of fixed effects, including the combination of season of farrowing and farrowing facilities (eight levels), parity (four levels), strain of boar (two levels) and the (linear) regression on *F*, **a** is the vector of additive genetic effects, **p** is the vector of permanent environmental effects associated with the sows, **e** is the vector of random residual effects, and **X**, **Z**_1_, and **Z**_2_ are incidence matrices relating fixed and random effects to observations. The expectation of **y** was assumed to be E**[y] = X*β***, and the variances and covariances of the random effects were assumed to be V(**a**) = **A***σ*^2^_a_, V(**p**) = **I**_m_*σ*^2^_p_ and V(**e**) = **I**_n_*σ*^2^_e_, where **A** is the pedigree-based numerator relationship matrix of order *N* (number of animals in the pedigree), **I**_m_ and **I**_n_ are identity matrices of order *m* (number of sows with litter size records), and *n* (number of records), respectively, and *σ*^2^_a_, *σ*^2^_p_, and *σ*^2^_e_ are the variances of additive genetic effects, permanent environmental effects, and residual effects, respectively. The analyses were performed using the REML/VCE 6.0 [14] and PEST [15] softwares.

Different analyses were performed by using the different inbreeding coefficients in the model (*F*_*ped*_, *F*_*snp*_, *F*_*roh*_, *F*_*roh_short*_ and *F*_*roh_long*_). The analysis using *F*_*ped*_ was carried out using all available performance and pedigree records, which included 823 sows with reproductive records on 2712 litters and a total pedigree file with 1032 animals. Analyses using *F*_*snp*_ and *F*_*roh*_ included only records for the 109 genotyped females and used estimates of variance and covariance components obtained from the *F*_*ped*_ analysis. Three analyses were implemented with the genomic inbreeding coefficients: (i) using *F* coefficients across the whole genome; (ii) using *F* coefficients for each autosome; and (iii) using *F* coefficients for specific regions within chromosomes.

### Ethical statement

The current study was carried out under a Project License from the INIA Scientific Ethic Committee. Animal manipulations were performed according to the Spanish Policy for Animal Protection RD1201/05, which meets the European Union Directive 86/609 about the protection of animals used in experimentation. We hereby confirm that the INIA Scientific Ethic Committee, which is the named IACUC for the INIA, specifically approved this study.

## Results

**Figure 1:**
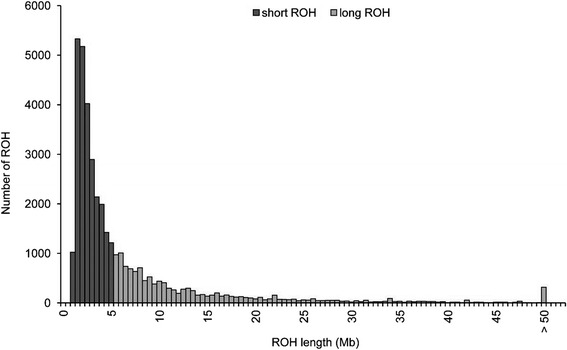
**Distribution of short and long ROH.**

**Figure 2:**
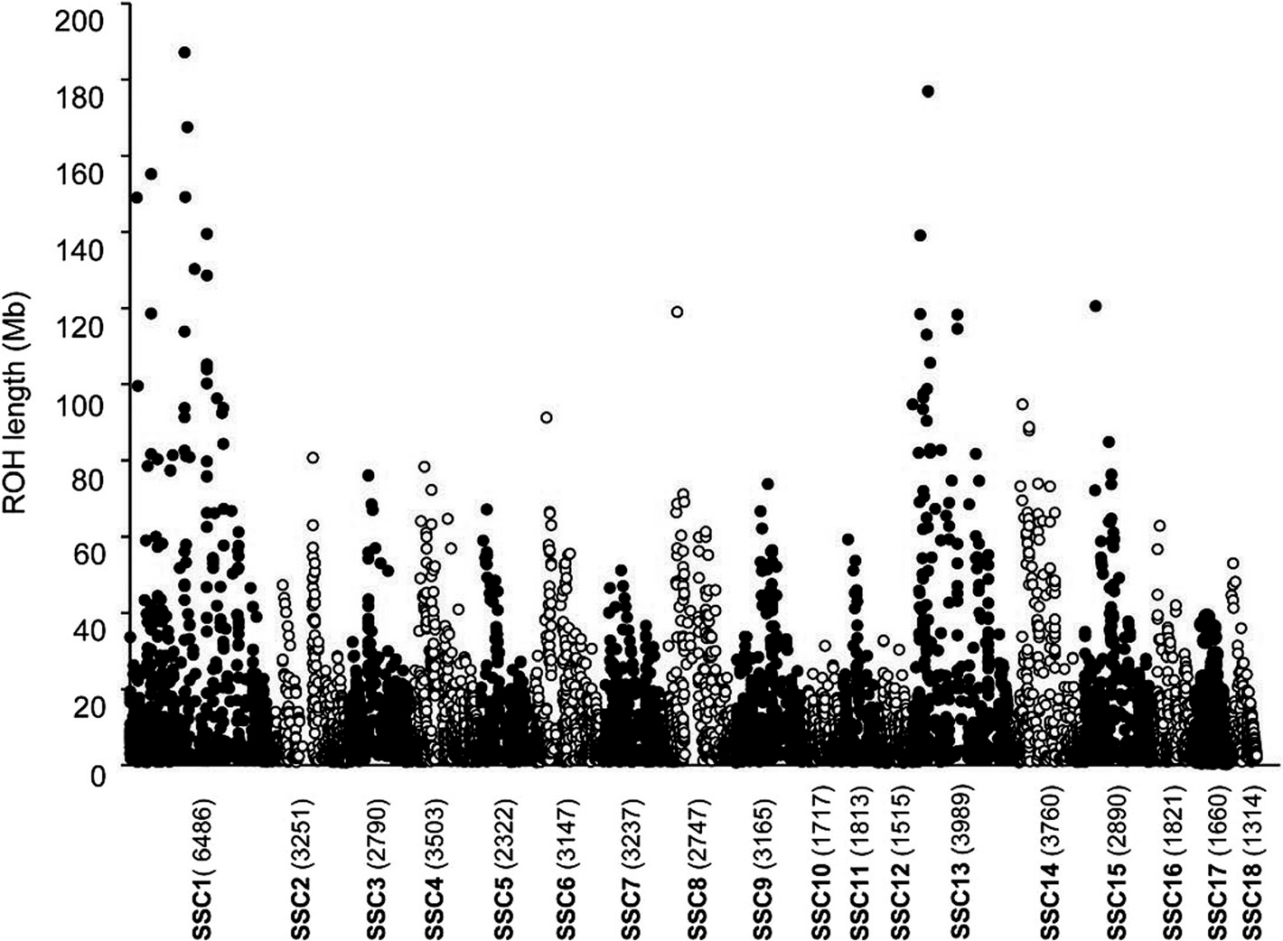
**Distribution of ROH according to their size within each autosome.** The number of SNPs per autosome is indicated in brackets.

Descriptive statistics for *F*_*ped*_, *F*_*snp*_, *F*_*snp_r*_ and *F*_*roh*_ are summarized in Table 1. The average *F*_*snp_r*_ was very high but this is simply due to an effect of scale since *F*_*snp_r*_ is not corrected for homozygosity in the base population and therefore includes IBS. The average inbreeding coefficient based on long ROH was very close to *F*_*ped*_, whereas the average *F*_*roh_short*_ was about four times lower than the average *F*_*ped*_. Although short ROH were more abundant (about double the number) than long ROH (Figure 1), their total contribution to the autosomal genome was relatively small (11.1% for short versus 34.2% for long ROH). Chromosomes SSC1 (SSC for *Sus scrofa*) and SSC13 contained the longest ROH, with sizes greater than 170 Mb, while the maximum size for ROH in the other autosomes was 120 Mb (Figure 2). This was as expected, given the negative correlation between physical (Mb) chromosome size and recombination rate in pigs [16]. Fisher [17] noted that the expected length of a DNA segment that is IBD follows an exponential distribution with mean equal to 1/2 *g* Morgans, where *g* is the number of generations since the common ancestor. Given this, large ROH (>5 Mb) reflect the expected inbreeding from a common ancestor that lived less than 10 generations ago. In our data, the average *F*_*roh_long*_ was about three times as large as the average *F*_*roh_short*_, thus suggesting that most inbreeding was recent.

**Table 1:** **Mean, standard deviation (SD) and range (Min, Max) of pedigree and SNP-derived inbreeding coefficients**

	**Mean**	**SD**	**Min**	**Max**
*F*_*ped*_	0.390	0.037	0.332	0.497
*F*_*snp_r*_	0.868	0.011	0.845	0.910
*F*_*snp*_	−0.046	0.091	−0.463	0.251
*F*_*roh*_	0.453	0.048	0.337	0.629
*F*_*roh_short*_	0.111	0.014	0.081	0.198
*F*_*roh_long*_	0.342	0.056	0.162	0.535

*F*_*ped*_ = pedigree-based inbreeding; *F*_*snp_r*_ *=* genomic SNP-by-SNP inbreeding estimated as the proportion of homozygous genotypes; *F*_*snp*_ *=* genomic SNP-by-SNP inbreeding based on the excess of SNP homozygosity; *F*_*roh*_ *=* genomic inbreeding based on all ROH; *F*_*roh_short*_ *=* genomic inbreeding based on short ROH; *F*_*roh_long*_ *=* genomic inbreeding based on long ROH.

Pearson correlations between the different inbreeding coefficients across individuals were positive and high (Table 2), except for correlations with *F*_*roh_short*_, which were smaller and negative. These negative correlations should be interpreted with caution because, although short ROH are likely to have originated from remote common ancestors, they could be covered up or included in some of the longer ROH. The high positive correlations of *F*_*ped*_ and *F*_*snp*_ (which are measures of overall inbreeding) with *F*_*roh_long*_ also support most of the inbreeding to be recent. Correlations computed for each autosome followed the same pattern as those computed for the whole genome [See Additional file 1]. In magnitude, the highest positive correlations across autosomes were consistently those between *F*_*snp*_ and *F*_*roh_long*_ and the highest negative correlations were those between *F*_*snp*_ and *F*_*roh_short*_.

**Table 2:** **Pearson correlations (SE) between different inbreeding coefficients measured at the whole-genome level**

	***F***_***ped***_	***F***_***snp***_	***F***_***roh***_	***F***_***roh_short***_
*F*_*snp*_	0.662 (0.051)			
*F*_*roh*_	0.631 (0.053)	0.968 (0.017)		
*F*_*roh_short*_	−0.241 (0.066)	−0.499 (0.059)	−0.450 (0.061)	
*F*_*roh_long*_	0.603 (0.054)	0.959 (0.019)	0.974 (0.015)	−0.641 (0.052)

*F*_*ped*_ = pedigree-based inbreeding; *F*_*snp*_ = genomic SNP-by-SNP inbreeding based on the excess of SNP homozygosity; *F*_*roh* =_ genomic inbreeding based on all ROH; *F*_*roh_short*_ = genomic inbreeding based on short ROH; *F*_*roh_long*_ = genomic inbreeding based on long ROH.

As indicated above, estimates of genetic parameters were obtained from the pedigree-based analysis and then used in all the subsequent analyses that used genomic inbreeding coefficients. Estimates of heritability (0.052 ± 0.025 for NBA and 0.077 ± 0.029 for TNB) and permanent environmental coefficients (0.073 ± 0.023 for NBA and 0.068 ± 0.024 for TNB) were of the same order of magnitude as those previously reported for Iberian pigs [18]. These estimates led to a repeatability estimate of 0.15 for both traits. The estimated genetic correlation between traits was very high (0.964 ± 0.024) and also close to previous estimates [19,20].

**Figure 3:**
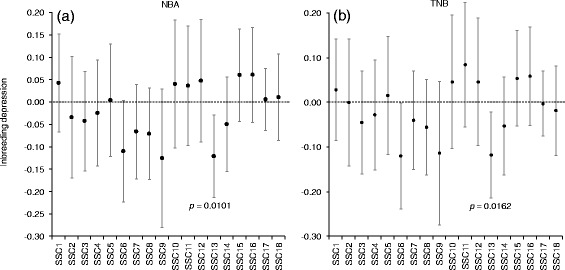
**Inbreeding depression estimates expressed as the change in phenotypic mean per 10% increase in** ***F***_***snp***_ **and 95% confidence intervals across autosomes for number of piglets born alive (NBA) (a) and total number of piglets born (TNB) (b).**

A significant reduction in both NBA and TNB with increases in the inbreeding coefficient was observed when performing the pedigree-based analysis using all available data (i.e. using records from 823 sows). Estimates of inbreeding depression were −0.197 ± 0.092 for NBA and −0.211 ± 0.104 for TNB per 10% increase in *F*_*ped*_. Although the effect of genomic *F* at the whole-genome level (i.e., using *F*_*snp*_ and *F*_*roh*_) with litter size was not significant (−0.267 ± 0.186 and −0.253 ± 0.194 for NBA and TNB, respectively, when using *F*_*snp*_, and −0.415 ± 0.374 and −0.335 ± 0.391 for NBA and TNB, respectively, when using *F*_*roh*_), significant (p < 0.05 for both traits, see Table 3) inbreeding depression was found for both traits when the genomic analyses were carried out at the autosomal level for SSC13. Estimates of inbreeding depression that were obtained from the analyses performed for each autosome using *F*_*snp*_ are presented in Figure 3 for NBA and TNB. Only SSC13 showed a significant effect. The estimated inbreeding depression for this chromosome was −0.121 ± 0.047 and −0.117 ± 0.049 per 10% increase in *F*_*snp*_ for both NBA and TNB, respectively.

**Table 3:** **Estimates of inbreeding depression on SSC13 for NBA and TNB**

	**NBA mean (SE)**	**TNB mean (SE)**
*F*_*snp*_	−0.121 (0.047)*	−0.117 (0.049)*
*F*_*roh*_	−0.230 (0.087)**	−0.231 (0.091)*
*F*_*roh_short*_	0.340 (0.380)	0.562 (0.395)
*F*_*roh_long*_	−0.181 (0.074)*	−0.188 (0.077)*

NBA = number of piglets born alive; TNB = total number of piglets born; results are expressed as the change in phenotypic mean per 10% increase in *F*; *F*_*snp*_ = genomic SNP-by-SNP inbreeding based on the excess of SNP homozygosity; *F*_*roh*_ = genomic inbreeding based on all ROH; *F*_*roh_short*_ = genomic inbreeding based on short ROH; *F*_*roh_long*_ = genomic inbreeding based on long ROH; significant values are indicated with asterisk (*p < 0.05, **p < 0.01).

Reductions in the number of piglets per 10% increase in *F*_*snp*_, *F*_*roh*_ and *F*_*roh_long*_ for SSC13 were all significant (see Table 3) and of the same order of magnitude as those derived from the pedigree-based analyses, which were based on the entire genome. There was no significant effect of *F*_*roh_short*_ on either NBA or TNB. This may be explained by purging of deleterious alleles in ancient generations, or by a bottleneck.

In order to detect specific genomic regions that cause inbreeding depression, all autosomes were fragmented in segments of equal size (three, five and eight segments per autosome) and three additional analyses per chromosome were carried out using *F*_*snp*_. When autosomes were divided into three segments, inbreeding depression was only significant for the first region of SSC13 (0.0 - 73 Mb) for both traits (Table 4). When they were divided in five segments, significance was found for the first (0–44 Mb) and the second (44–88 Mb) regions of SSC13 for both traits. Finally, when autosomes were divided into eight fragments, only the second region of SSC13 showed a significant result for both traits. This region is 32.4 Mb in size and is located between 27 and 55 Mb. No other regions showed a significant result for any of the traits.

**Table 4:** **Estimates of inbreeding depression for different regions of SSC13 for NBA and TNB**

**Region**	**Region**	**position**	**NBA**		**TNB**	
	**Start (Mb)**	**End (Mb)**	**Mean (SE)**	***p*** **value**	**Mean (SE)**	***p*** **value**
**SSC13 divided in three segments**						
I	0.09	72.92	−0.138 (0.039)	0.0004^***^	−0.144 (0.041)	0.0004^***^
II	72.98	145.73	−0.035 (0.028)	0.2053	−0.033 (0.029)	0.2526
III	145.77	218.59	−0.040 (0.038)	0.2915	−0.032 (0.040)	0.4190
**SSC13 divided in five segments**						
I	0.09	43.77	−0.131 (0.041)	0.0013^**^	−0.135 (0.043)	0.0016^**^
II	43.88	87.38	−0.066 (0.025)	0.0092^**^	−0.071 (0.026)	0.0070^**^
III	87.58	131.18	−0.039 (0.023)	0.0859	−0.038 (0.023)	0.1071
IV	131.20	174.85	−0.031 (0.036)	0.3900	−0.016 (0.038)	0.6669
V	175.01	218.59	−0.019 (0.035)	0.5749	−0.020 (0.036)	0.5914
**SSC13 divided in eight segments**						
I	0.09	27.35	−0.046 (0.046)	0.3138	−0.077 (0.043)	0.1115
II	27.45	54.66	−0.072 (0.020)	0.0003^**^	−0.064 (0.020)	0.0017^**^
III	54.77	82.03	−0.029 (0.020)	0.1479	−0.039 (0.020)	0.0560
IV	82.03	109.17	−0.028 (0.022)	0.1967	−0.024 (0.023)	0.2901
V	109.42	136.65	−0.033 (0.023)	0.1431	−0.036 (0.024)	0.1220
VI	136.69	163.96	−0.022 (0.037)	0.5506	−0.005 (0.038)	0.9016
VII	164.33	191.28	−0.035 (0.023)	0.1297	−0.036 (0.024)	0.1252
VIII	191.36	218.59	0.003 (0.027)	0.8038	0.006 (0.028)	0.8277

NBA = number of piglets born alive; TNB = total number of piglets born; results are expressed as the change in phenotypic mean per 10% increase in *F*_*snp*_; significant values are indicated with asterisk (**p < 0.01, ***p < 0.001).

We also performed a bootstrap test by generating 1000 bootstrap replicates of the individual phenotypes and genotypes for the region that was significant in the inbreeding depression analysis. Bootstrap replicates were created by randomly resampling the individual phenotypes and inbreeding depression analyses were repeated for each replicate. When fragmenting SSC13 in eight segments, the average of the estimates for the effect across bootstrap replicates was 0, with a standard error that was lower than that obtained from the real data (0.020) in all cases except one. This is equivalent to a bootstrap p-value of 0.001. These results constitute more evidence that the signals detected are not spurious.

## Discussion

We have detected inbreeding depression associated with a specific region of SSC13 for two reproductive traits in a highly inbred strain of Iberian pigs using different measures of genome-wide inbreeding coefficients. It is important to note that the signal detected was significant despite the small sample size available for the study (109 sows). This indicates that it would be extremely rare that the effects detected here are spurious.

Also, our results are consistent with those obtained by Noguera et al. [19] who performed one of the first genome-wide scans for prolificacy traits in pigs. They used data from a Guadyerbas x Meishan F2 intercross using SNPs and microsatellite markers and detected a quantitative trait locus (QTL) on SSC13 for both NBA and TNB. This QTL region extends from about 38 to 194 Mb and overlaps with the region identified here. Specifically, the region detected in the inbreeding depression analysis is shorter (it spans from 27 to 54 Mb) and overlaps with the first part of the QTL region detected by Noguera et al. [19]. We examined the gene content of this common region by using the porcine genome annotation *Sscrofa*10.2 in BioMart tool of Ensembl (ensembl.org/biomart) and the Ensembl Genes 69 database and found 271 annotated genes. Interestingly three of these genes, the *inter-alpha-trypsin inhibitor heavy chains 1, 3* and *4* (*ITIH-1, ITIH-3* and *ITIH-4*) map to 38 Mb on SSC13 and play several important roles in maintaining the uterine surface glycocalyx during placental attachment in pigs [20]. Moreover, these genes have been previously associated with NBA and TNB [21]. Specifically, using the same material as Noguera et al. [19], Balcells et al. [21] analyzed the porcine *ITIH-1, −3* and −*4* gene sequences in order to identify polymorphisms that could explain differences in prolificacy of sows. Their results revealed significant associations with NBA and TNB for two SNPs in *ITIH-1*, four SNPs in *ITIH-3*, and four SNPs in *ITIH-4*. Thus, the studies of Noguera et al. [19] and Balcells et al. [21] support our findings, since genes that affect both NBA and TNB are located in the region identified on SSC13. Another recent whole-genome association study identified QTL regions for NBA and TNB that partially overlapped with the region identified here [22].

Several studies that compared the same inbreeding coefficients as used here indicated that *F*_*roh*_ is a better measure of IBD than *F*_*snp*_ [4,5,23]. However, Keller et al. [4] showed that as *N*_*e*_ decreases, the similarity of both measures of molecular inbreeding (*F*_*roh*_ and *F*_*snp*_) increases. Based on this and going down to an *N*_*e*_ of 10 (the estimate for this herd [3]), we can expect that the measures of *F*_*snp*_ and *F*_*roh*_ will be highly correlated (0.97 ± 0.02). In fact, the correlation between *F*_*ped*_ and *F*_*roh*_ found in our study was similar to that between *F*_*ped*_ and *F*_*snp*_ (0.63 ± 0.05 and 0.66 ± 0.05, respectively) All this information validates the use of *F*_*snp*_ as a measure of IBD in this population, and therefore, as a suitable coefficient to perform the inbreeding depression analyses.

Previous studies aimed at detecting inbreeding depression using SNPs have focused on the whole genome level [6,23-25]. These include human studies that investigated the association between inbreeding and particular diseases [5,11,13,26,27]. Only a few of these have attempted to identify specific genomic regions that cause depression [11,13,27]. Keller et al. [8] used a ROH mapping approach to analyze the association of *F*_*roh*_ with schizophrenia risk. The approach consisted of dividing the autosomes in a large number of segments of equal size and performing regressions of disease status on whether or not individuals had a ROH in each segment. They found significant associations between specific genomic regions and disease status. Recently Pryce et al. [28] have followed a similar approach for detecting inbreeding depression for fertility and milk production traits in dairy cattle.

In order to compare our approach for detecting genomic regions associated to inbreeding depression using *F*_*snp*_ with the approach of Keller et al. [8], we divided the autosomes in segments of approximately 2 Mb and recorded for presence or absence of ROH within each segment. The results from these regressions showed a significant effect in the same region on SSC13 as detected with the analysis using *F*_*snp*_ [See Additional file 2]. Although this significant effect was lost when multitest correction was applied, the results support our previous finding. Additional file 2 shows results for NBA but the same pattern was found for TNB. It should be noted that our analysis using a continuous variable (i.e., *F*_*snp*_) instead of a dichotomous variable (i.e., presence/absence of ROH) has more power to detect associations between phenotypes and inbreeding within specific regions.

We also performed an association analysis for the region involved in inbreeding depression. It was conducted using the same data (265 phenotypic records on 109 sows) and statistical model as described above for inbreeding depression analyses but also included the SNP genotype as a fixed effect. Each SNP was tested separately for association with the trait and both additive and dominant effects were estimated. We found several SNPs that showed significant dominance effects and non-significant additive effects for both traits (Additional file 3 shows the results for NBA only but similar results were found for TNB [See Additional file 3]). This result is consistent with the findings from the inbreeding depression analyses and agrees with directional dominance for the genes involved.

## Conclusions

In summary, using genome-wide SNP information, we detected inbreeding depression in a specific region that contains genes associated with litter size in the isolated population of Guadyerbas Iberian pigs. The availability of dense SNP platforms has created opportunities to estimate homozygosity without using pedigree relationships and to obtain patterns of homozygosity along an individual’s genome. Our results highlight the importance of SNP chips for providing new insights into where genes causing inbreeding depression are located in the genome and thus, offer a complementary tool to QTL analysis for mapping studies.

## Additional files

Additional file 1:
**Pearson correlations between different inbreeding coefficients across autosomes.** *F*_*ped*_ = pedigree-based inbreeding; *F*_*snp*_ = genomic SNP-by-SNP inbreeding based on the excess of SNP homozygosity; *F*_*roh_short*_ = genomic inbreeding based on short ROH; *F*_*roh_long*_ = genomic inbreeding based on long ROH. Additional file 2:
**Inbreeding depression estimates in SSC13 expressed as the change in phenotypic mean per 10% increase in** ***F***_***roh***_ **and 95% confidence intervals for number of piglets born alive.** Results derived from the inbreeding depression analyses based on the presence/absence of a ROH when chromosomes were divided into segments of approximately 2 Mb. Additional file 3:
**Estimates of dominance effects and 95% confidence intervals in the inbreeding depression region for number of piglets born alive.** Results derived from the association analysis for the detected region involved in inbreeding depression.
