# Supplementary figures and images for: Detecting inbreeding depression for reproductive traits in Iberian pigs using genome-wide data
Source: Genet Sel Evol. 2015 Jan 17;47(1):1. doi: 10.1186/s12711-014-0081-5 (PMC4297446; doi:10.1186/s12711-014-0081-5)

■  $F_{gen} - F_{roh\_long}$     \*  $F_{gen} - F_{snp}$   
 △  $F_{gen} - F_{roh\_short}$     □  $F_{snp} - F_{roh\_long}$     ▲  $F_{snp} - F_{roh\_short}$

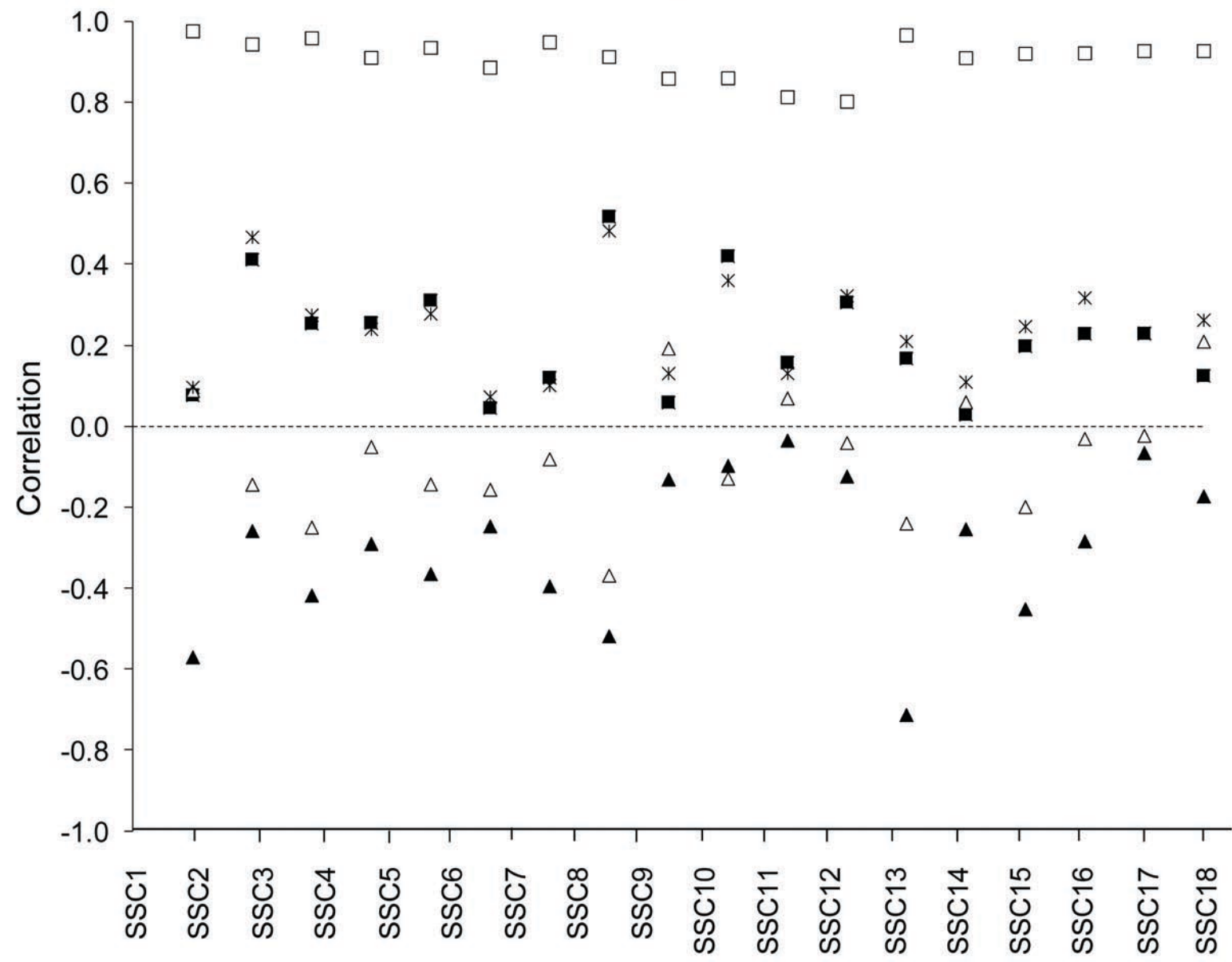

Supplement: Additional file 1: — Pearson correlations between different inbreeding coefficients across autosomes. F ped = pedigree-based inbreeding; F snp = genomic SNP-by-SNP inbreeding based on the excess of SNP homozygosity; F roh_short = genomic inbreeding based on short ROH; F roh_long = genomic inbreeding based on long ROH. [file 12711_2014_81_MOESM1_ESM.pdf]

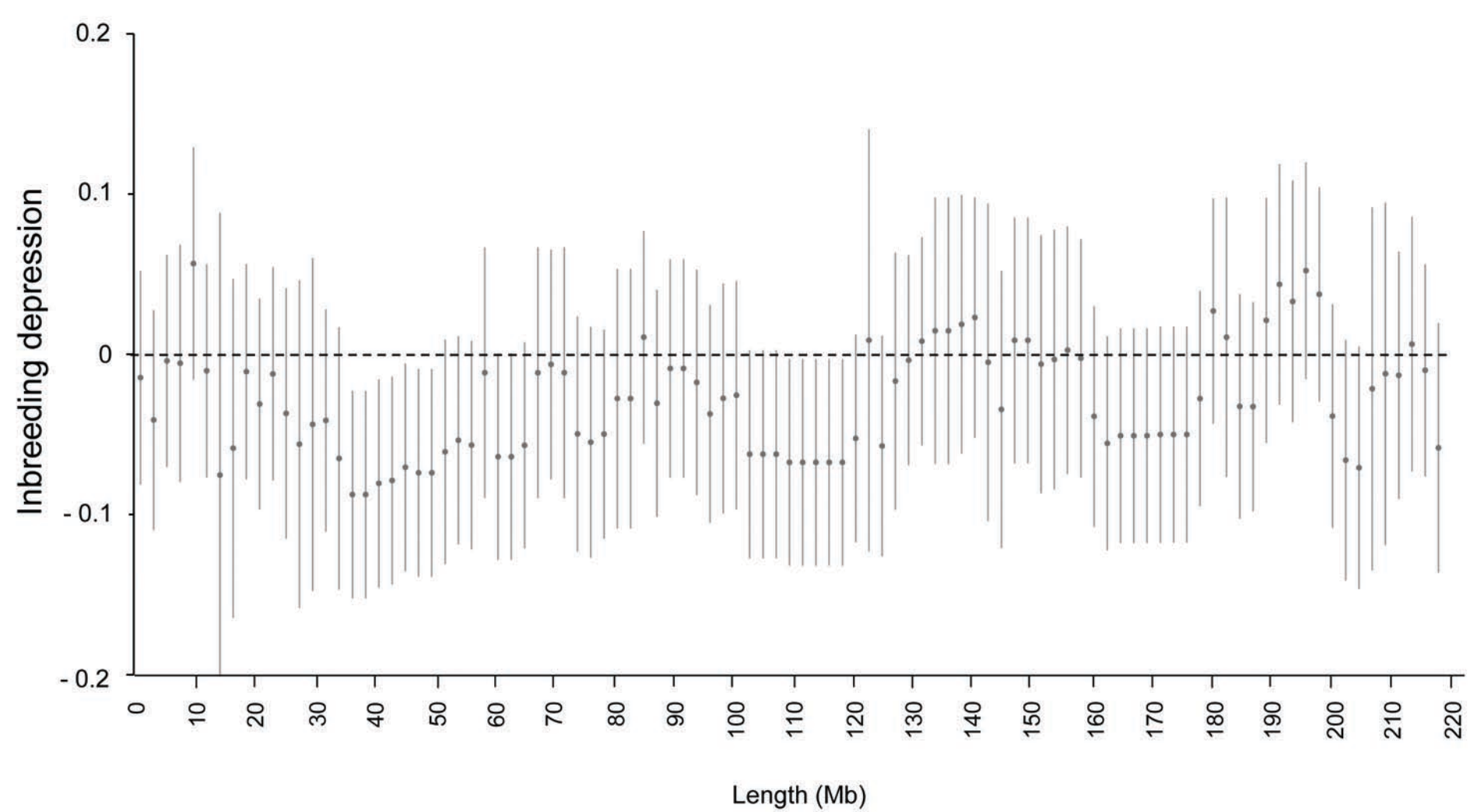

Supplement: Additional file 2: — Inbreeding depression estimates in SSC13 expressed as the change in phenotypic mean per 10% increase in F roh and 95% confidence intervals for number of piglets born alive. Results derived from the inbreeding depression analyses based on the presence/absence of a ROH when chromosomes were divided into segments of approximately 2 Mb. [file 12711_2014_81_MOESM2_ESM.pdf]

Dominance effect

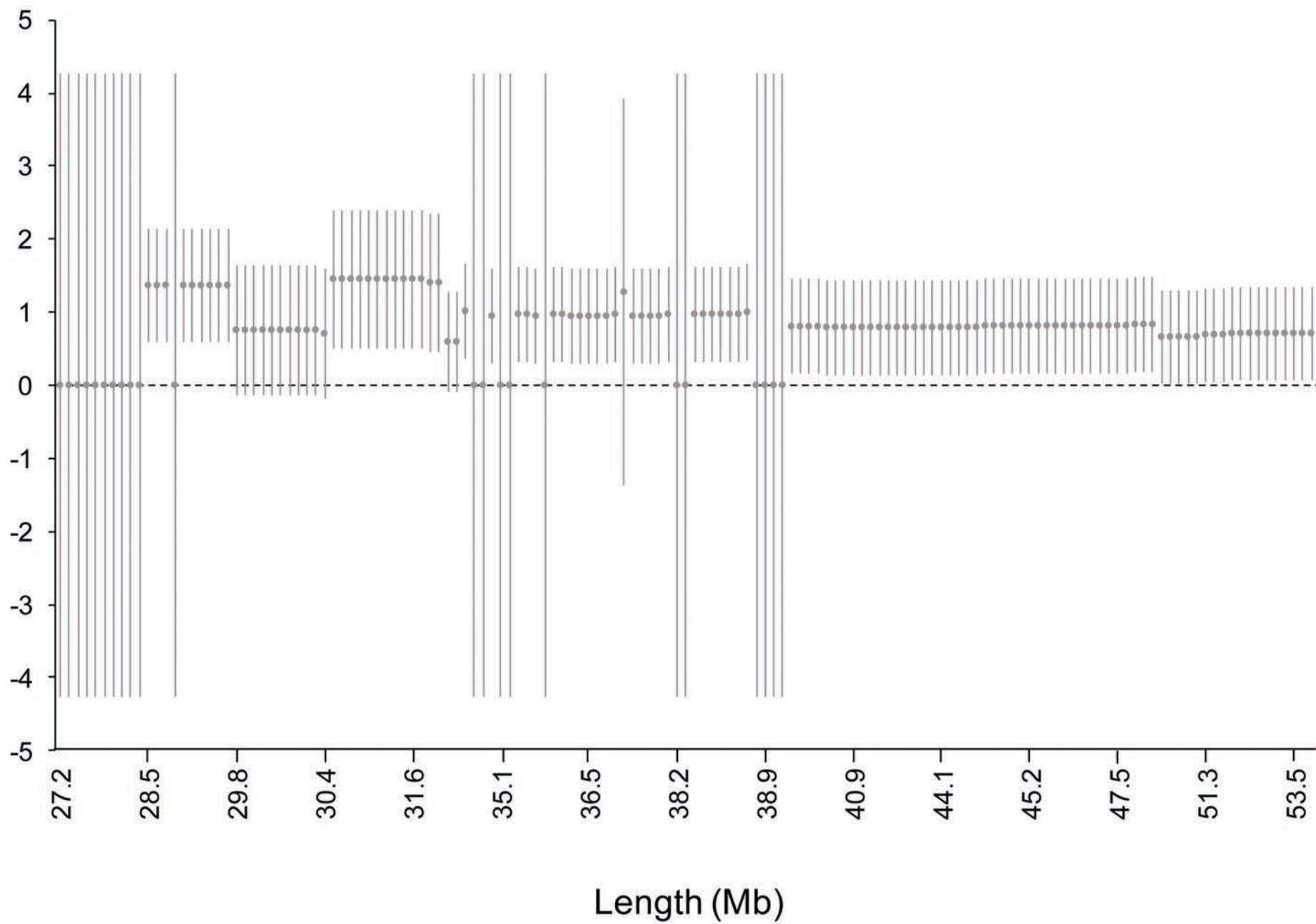

Supplement: Additional file 3: — Estimates of dominance effects and 95% confidence intervals in the inbreeding depression region for number of piglets born alive. Results derived from the association analysis for the detected region involved in inbreeding depression. [file 12711_2014_81_MOESM3_ESM.pdf]
